# Bilateral oropharyngeal hairy polyps: a rare cause of dyspnea in newborns^☆^

**DOI:** 10.1016/j.bjorl.2015.06.001

**Published:** 2015-10-16

**Authors:** Rasim Yilmazer, Burak Kersin, Erkan Soylu, Gokhan Altin, Asli Cakir, Fahrettin Yilmaz

**Affiliations:** Istanbul Medipol University, Department of Otorhinolaryngology, Istanbul, Turkey

## Introduction

Hairy polyp (HP) is one of the causes of congenital dyspnea, a rare developmental malformation of bigerminal origin that comprises both ectodermal and mesodermal elements foreign to the site in which it is found. It typically presents as a pedunculated mass in the oropharynx and nasopharynx. Major symptoms at presentation are related with respiratory obstruction and feeding problems. In the literature, congenital pharyngeal hairy polyps are generally unilateral.^1^, ^2^

## Case report

**Figure 1:**
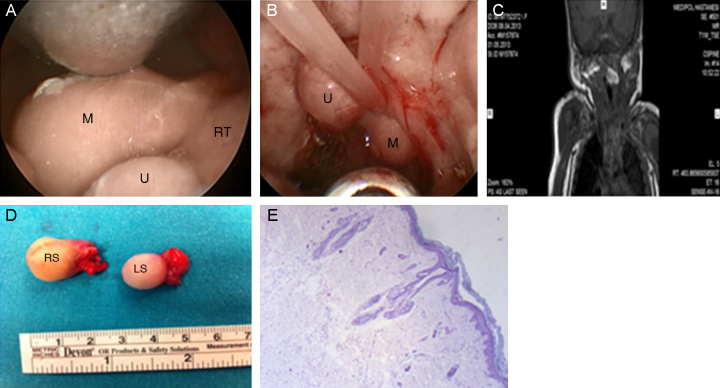
(A) Hairy polyp originating from the posterior pillar of the right tonsil (M, mass; U, uvula; RT, right tonsil). (B) Hairy polyp originating from the posterior pillar of the left tonsil elongating through the nasopharynx (M, mass; u, Uvula). (C) Magnetic resonance image of the hairy polyp originating from the posterior pillar of the right tonsil. (D) The excised specimens (RS, right specimen; LS, left specimen). (E) Keratinized squamous epithelium on the surface and mesenchymal tissue components, such as hair follicles lining the fibroadipose core (HE 40×).

A 3440 g white female newborn was born by spontaneous vaginal delivery at 39 weeks gestation of a 20 year-old G2P1A1 female, following a normal pregnancy. Immediately at birth, she had respiratory distress and cyanosis, and then required oropharyngeal intubation and mechanical ventilation. On oropharyngeal examination, there was only one soft, skin-covered mass originating from the posterior pillar of the right tonsil and elongating to the level of the oropharynx; however, on nasopharyngoscopy, another similar mass originating from the posterior pillar of the left tonsil was visualized, elongating through the nasopharynx (Fig. 1A and B). Additionally, bilateral low-set ear anomaly was found. Magnetic resonance imaging (MRI) of the neck demonstrated a well-defined, hyperintense polypoid masses attaching to posterior side of the tonsils (Fig. 1C). MRI features of the mass lesions were defined as heterogeneous hyperintense appearance on T1 and T2-weighted series. In fat-suppressed sequences, it was suppressed, and in the post-contrast series, there was no significant contrast enhancement.

Under general anesthesia, HPs were completely removed through transoral route under endoscopic visualization by cold dissection and bipolar electrocautery (Fig. 1D). Pathological examination showed two HPs, 27 mm × 18 mm × 14 mm and 23 mm × 13 mm × 13 mm in size (Fig. 1E). She was discharged after two days. Unfortunately, after the first follow-up, the patient was lost to follow-up and thus it was not possible to determine whether she had a syndrome.

## Discussion

The most common localization of HP is from the nasopharynx; arising from the superior aspect of the soft palate or lateral pharyngeal wall. Female infants are six times more likely to be affected than males.^3^

The most common symptom of the HP is respiratory distress. Airway obstruction may occur if the polyp becomes impacted in the larynx, and deaths have been reported.^1^ Differential diagnosis of the pharyngeal obstruction in a newborn includes choanal atresia, intranasal glioma, and encephalocele.^3^ Since HP is rare and may present with respiratory distress in newborns, it should be considered in differential diagnosis, and a careful endoscopic evaluation by otolaryngologists must be performed.

There are few multiple bigerminal (HPs) and trigerminal teratomas that have been reported in the head and neck, and within the airway.^2^ Morgan^4^ reported a male newborn with two HPs that originated from the left side of the oropharynx and nasopharynx. Since the laterality of the nasopharyngeal mass was not noted, it was not clear whether they were bilateral. Franco et al.^5^ reported a 58 year-old female with bilateral oropharyngeal HPs. She was referred to them because of feeding difficulties and mild dyspnea, but she had had no relevant medical history. Hence, they are probably not congenital. To the best of the authors’ knowledge, this is the first case of bilateral pharyngeal HPs in a newborn in the literature. A thorough examination of the oronasopharynx is mandatory in newborns with an oropharyngeal mass in order to avoid overlooking a second mass.

The endoscopic approach aids avoidance of injury to the eustachian tube orifice, as well as excising the pathologic lesions more completely. However, velopharyngeal dysfunction is observed as a complication of surgical approach.^6^

## Final remarks

Since HPs can be bilateral, diagnosis requires a complete otorhinolaryngological examination. Transoral endoscopic resection should be performed to avoid undesirable complications.

## Conflicts of interest

The authors declare no conflicts of interest.
